# A Healthy Brain in a Healthy Body: Brain Network Correlates of Physical and Mental Fitness

**DOI:** 10.1371/journal.pone.0088202

**Published:** 2014-02-03

**Authors:** Linda Douw, Dagmar Nieboer, Bob W. van Dijk, Cornelis J. Stam, Jos W. R. Twisk

**Affiliations:** 1 Department of Neurology, Vrije Universiteit Medical Center, Amsterdam, The Netherlands; 2 Department of Anatomy and Neurosciences, Vrije Universiteit Medical Center, Amsterdam, The Netherlands; 3 Department of Epidemiology and Biostatistics, Vrije Universiteit Medical Center, Amsterdam, The Netherlands; 4 Department of Clinical Neurophysiology and Magnetoencephalography Center, Vrije Universiteit Medical Center, Amsterdam, The Netherlands; 5 Department of Epidemiology and Biostatistics, EMGO Institute for Health and Care Research, VU University Medical Center, Amsterdam, the Netherlands; University of Namur, Belgium

## Abstract

A healthy lifestyle is an important focus in today's society. The physical benefits of regular exercise are abundantly clear, but physical fitness is also associated with better cognitive performance. How these two factors together relate to characteristics of the brain is still incompletely understood. By applying mathematical concepts from ‘network theory’, insights in the organization and dynamics of brain functioning can be obtained. We test the hypothesis that neural network organization mediates the association between cardio respiratory fitness (i.e. VO_2_ max) and cognitive functioning. A healthy cohort was studied (n = 219, 113 women, age range 41–44 years). Subjects underwent resting-state eyes-closed magneto-encephalography (MEG). Five artifact-free epochs were analyzed and averaged in six frequency bands (delta-gamma). The phase lag index (PLI) was used as a measure of functional connectivity between all sensors. Modularity analysis was performed, and both within and between-module connectivity of each sensor was calculated. Subjects underwent a maximum oxygen uptake (VO_2_ max) measurement as an indicator of cardio respiratory fitness. All subjects were tested with a commonly used Dutch intelligence test. Intelligence quotient (IQ) was related to VO_2_ max. In addition, VO_2_ max was negatively associated with upper alpha and beta band modularity. Particularly increased intermodular connectivity in the beta band was associated with higher VO_2_ max and IQ, further indicating a benefit of more global network integration as opposed to local connections. Within-module connectivity showed a spatially varied pattern of correlation, while average connectivity did not show significant results. Mediation analysis was not significant. The occurrence of less modularity in the resting-state is associated with better cardio respiratory fitness, while having increased intermodular connectivity, as opposed to within-module connections, is related to better physical and mental fitness.

## Introduction

A healthy lifestyle is a major focus in today's society. Regular exercise and adequate physical fitness have proven to be important for the immune system, metabolism, prevention of infectious disease, skeletal functioning, and risk of cancer [1]–[6]. In addition to these physical benefits, cardiorespiratory fitness is also related to better cognitive functioning [7]. Several neural factors have been reported to mediate the relationship between mental and physical fitness, including increased neural vascularization [8], increased production of brain derived neurotrophic factor (BDNF; [9]), increased hippocampal volume [10], and higher levels of N-acetylaspartate [11], although none of these mediators fully explain the reported associations.

Another framework that has elucidated the neural correlates of the association between cognition and physical fitness is resting-state functional connectivity, as measured with functional magnetic resonance imaging (fMRI). The resting-state, during which no task is present and alert relaxation is achieved, can be characterized by several highly robust networks [12], [13], of which the default mode network (DMN) is the most stable and best studied example [14], [15]. This network seems to be the functional backbone of the brain [16], and is implicated in almost all neurological diseases. With respect to cardiorespiratory fitness, higher connectivity within the DMN (as measured by seeding the posterior cingulate cortex and examining its significantly correlated regions) is associated with better fitness level, and DMN connectivity mediates the association between physical fitness and cognitive functioning [17]. Moreover, a one-year aerobic training intervention in older adults improves functional connectivity within several resting-state networks, including the DMN and the fronto-parietal network, which is thought to be important for working memory [18]. Conversely, overweight adults show increased DMN connectivity, which normalizes after a six month exercise program [19]. The important role of functional connectivity in the relationship between physical fitness and cognition was confirmed in another study by Voss and colleagues, showing that the association between exercise and connectivity is related to BDNF, insulin-like growth factor type 1 (IGF-1), and vascular endothelial growth factor (VEGF), which are markers for neuroplasticity [20].

However, fMRI is an indirect measure of neural functioning, as it measures the slowly operating process of blood oxygenation. Functional connectivity can also be determined frommagnetoencephalography (MEG), which is a much more direct measure of neural activity. Furthermore, functional connectivity in general can be used as a starting point for more extensive, higher-order analysis of the entire brain network using graph theory [21]–[23]. This type of analysis has shown that the brain network is very comparable to many simpler biological and sociological systems [24]. This elegantly theory-governed but still data-driven property has made the application of network theory to the brain a very interesting endeavor. For instance, the brain network is a ‘small-world’, combining local segregation with global integration [24]–[27]. Brain network topology is to a large extent genetically determined [28]–[30] and is disturbed in several neurological diseases [21], [31].

The functional brain network also correlates with global cognitive functioning and intelligence [32], [33], indicating network theory may add relevant information on neural correlates of functioning above connectivity alone. Important information about the integrity of the (brain) network can be extracted by looking at modularity. Modules are clusters of nodes, or brain areas, that are highly connected to each other, but much less to nodes outside of their own module [34]. In the brain, five to seven modules can be discerned, which correspond to major functional systems [35]. Moreover, the role that specific brain areas play both within their module and in connecting other modules has proven relevant to brain functioning [36]–[38].

In this study, physical fitness, intelligence, and their neural correlates in terms of network modularityare investigated. We aimed to prove that VO_2_ max, a measure of cardio respiratory fitness,is related to modular network topology based on MEG in a large group of healthy subjects. Furthermore, we hypothesized that intelligence is associated with physical fitness mediated through brain network topology in terms of modular organization.

## Materials and Methods

### Ethics statement

This study was approved by the Medical Ethical Institutional Review Board of the VU University Medical Center. All subjects gave written informed consent before participation. This study was carried out in accordance with the Declaration of Helsinki.

### Subjects

All subjects participated in a prospective longitudinal study, originally investigating natural development of growth, health and lifestyle of adolescents, the Amsterdam Growth and Health Longitudinal Study (AGHLS). This cohort study started in 1976 with four annual measurements and continued with an extensive number of assessments with five to seven year time intervals[39]. All participants were born between 1961 and 1965 and were residents of the Netherlands. First- and second-year pupils from two equally large secondary schools were recruited. In 2006, MEG recordings of the remaining 344 participants (who were all between 41 and 44 years old) were obtained, in addition to the health parameters that were investigated at each time-point of the AGHLS [40]. These data are not publicly available at this point.

### Physical fitness

Physical fitness was measured with a maximal running test on a treadmill (Quinton 18–54; Quinton, Bothell, Wash), with a speed of 8 km/h and increasing slope (every 2 minutes) and with direct measurements of oxygen uptake (Ergoanalyzer; Jaeger, Bunnik, the Netherlands). Maximum oxygen consumption (VO_2_ max) was used as a measure of physical fitness (Kemper, 1995). This measurement was performed approximately six years before MEG recording.

### Cognitive performance

Subjects underwent a cognitive test battery at the time of MEG recording, to assess full-scale intelligence. The test battery administered included the shortened Groninger Intelligence Test (GIT [41]), which is a commonly used Dutch intelligence test. Three subtests of the entire GIT were used, constituting the short version of the test to assess intelligence [42]. Completion of the test took approximately 45 minutes per subject.

### Magnetoencephalography

Magnetic fields were recorded for five minutes while subjects were seated inside a magnetically shielded room (Vacuumschmelze GmbH, Hanau, Germany), using a 151-channel whole-head MEG system (CTF SystemsInc., Port Coquitlam, BC, Canada). A third-order software gradient was used after online band-pass filtering between 0.25 and 125 Hz. Sample frequency of recording was 625 Hz. Fields were measured during a no-task, eyes-closed condition of five minutes. At the beginning and ending of each recording, the head position relative to the coordinate system of the helmet was recorded by passing small alternating currents through three head position coils attached to the left and right pre-auricular points and the nasion on the subject's head.

For each subject, the first five artifact-free epochs of 4096 samples (6.554 s) were selected by one of the authors [BWvD]. All data analyses were performed using BrainWave [CJS, version 0.9.58, available from http://home.kpn.nl/stam7883/brainwave.html]. Before calculating connectivity and network topology, epochs were band-pass filtered into the commonly used frequency bands delta (0.5–4 Hz), theta (4–8 Hz), lower alpha (8–10 Hz), upper alpha (10–13 Hz), beta (13–30 Hz), and gamma (30–45 Hz). All further analyses were performed for these bands separately. The average relative power in the six abovementioned frequency bands was calculated in each subject using a fast Fourier transform as described in [43].

### Phase Lag Index (PLI)

As a measure of functional connectivity, the phase lag index (PLI) was used[44], which calculates the asymmetry of the distribution of (instantaneous) phase differences between two time-series. This asymmetry can be obtained from a time series of phase differences ΔΦ (t_k_), *k* = 1… *N* samples: 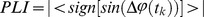



The phase difference Δφ is defined in the interval [−π, π] and <> denotes the mean value. Volume conduction causes a zero phase lag between two time-series, but the presence of a consistent, non-zero, phase lag between two time-series reflects true interactions that are unaffected by volume conduction or common sources.

### Modularity, between and within module connectivity

First, to describe modularity in the whole-brain network we used a version of previously described approaches[45], adapted for weighted networks [37], [46]: 
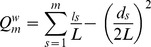
 where *m* is the number of modules, *l_s_* is the sum of the weights of all links in module *s*, *L* is the total sum of all weights in the network, and *d_s_* is the sum of the strength of all nodes (i.e. the summed weights per node) in module *s*. In short, the relation between intra- and intermodular connections determines the strength of each module. This measure describes modularity by summing the relative strength of all the network modules, which takes both within and between module connections into account. A strongly modular network has modularity value close to 1, while modularity is closer to 0 (but not absent) in a random network. Finding the optimal modular organization in a network is a computationally intensive problem. Simulated annealing can overcome part of this issue, and was used in the current study [45]. Initially, each of the *N* nodes was randomly assigned to one of m possible clusters, where the initial m was taken as the square root of *N*. At each step, one of the nodes was chosen at random, and assigned a different randomly chosen module number from the interval [1,*N*]. Modularity was calculated before and after this. The cost *C* was defined as 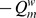. The new partitioning was preserved with probability *p*: if the final cost *C_f_* was lower or equal to the initial cost *C_i_* (indicating no added cost when preserving the partition), *p* was equal to 1. If *C_f_* was higher than *C_i_*, *p* was calculated as follows: 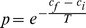



The temperature *T* was 1 initially, and was lowered once every 100 steps as follows: *T_new_* = 0.995 *T_old_*. In total, the simulated annealing algorithm was run for 10^6^ steps. The partition with the strongest modular organization (highest Q) was identified separately for each epoch of every person for all the different frequency bands, and subjected to further graph analysis.

**Figure 1:**
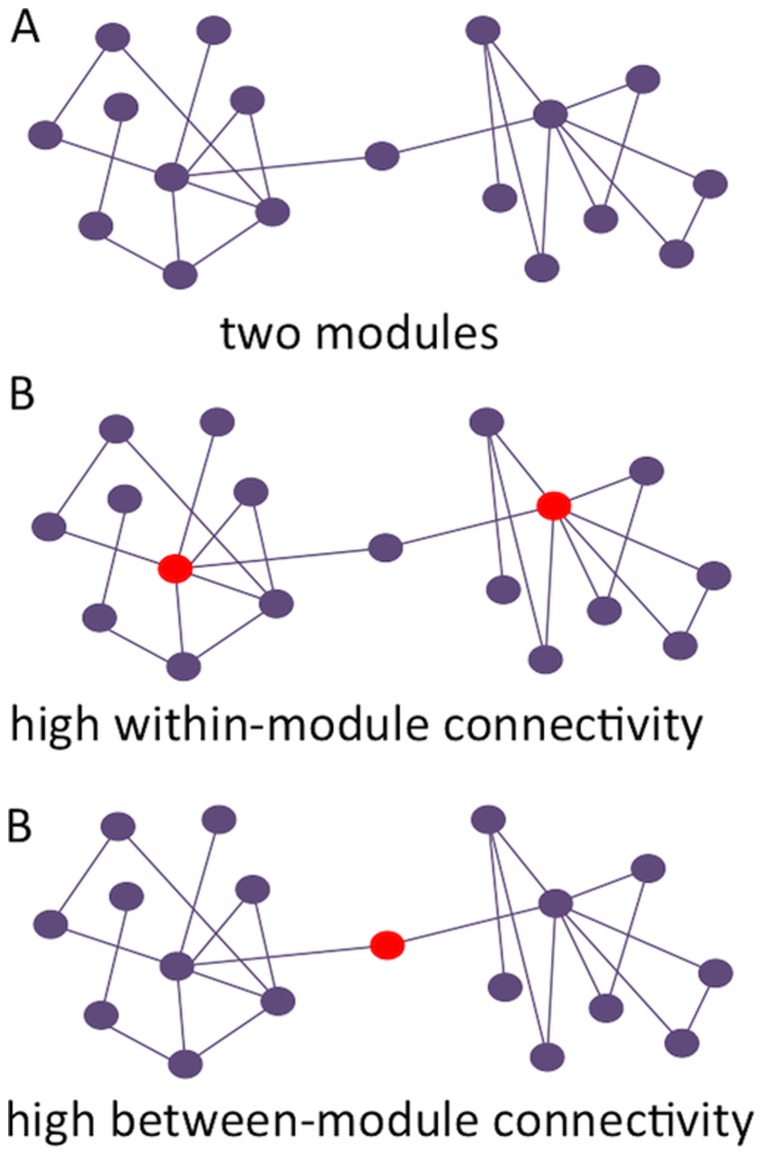
Schematic representation of modularity and modular connectivity. Note. In (a), two modules can be discerned. These modules show high within-module connectivity, but low between-module connectivity. (b) depicts two nodes in the network that are characterized by high within-module connectivity, while (c) shows a node with very high between-module connectivity.

Once the modular organization in a network has been determined, the topological role of individual nodes can be described in greater detail: nodes can be mainly involved in communication with other nodes in the same module, but can also preferably interact with other modules (see figure 1). This aspect is quantified by two properties: the within-module degree (Z_i_), and the participation coefficient (PC) [45]. The within-module degree measures the connectivity of the node within the module compared to the other nodes in the same module, and thus describes the relative importance within the module. It was defined as follows: 
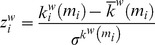





Here, *m_i_* is the module containing node *i*, 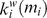 is the within module strength of node *i* (the sum of all weights of the links between *i* and all other nodes in *m_i_*), and 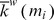 and 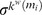 are the respective mean and standard deviation of the within-module m_i_ degree distribution.

The participation coefficient expresses how strongly a node is connected to other modules, and the weighted version is defined as: 
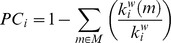



*M* is the set of modules, and 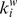 is the sum of all weights of the links between *i* and all nodes in module *m*. The within module degree and the participation coefficient determine the identity of a node in the modular network structure.

### Statistical analysis

Statistical analyses were performed using PASW Statistics package (version 20.0) and Matlab version r2012b. Differences between men and women regarding VO_2_max, IQ, and average head surface were tested using Student's t-tests. The association between intelligence and VO_2_ max was analyzed using a linear regression with IQ as the dependent variable and VO_2_ max as independent variable, adjusted for sex. The association between VO_2_ max and modular network topology was investigated with regression analysis in which modularity indices per frequency band were the dependent variables and VO_2_ max the dependent variable, adjusted for sex, head surface size, and relative power per frequency band. Similar analyses were used to explore average between-module connectivity. Finally, the relationships between VO_2_ max, network modularity, and intelligence were further studied using mediation analyses. Mediation analysis investigates whether a third parameter underlies an observed relationship between two variables, meaning that the third variable governs the association between the other two. In our study, we hypothesized that the association between VO_2_ max and intelligence is mediated by brain network modularity. This mediation model was tested using the INDIRECT PASW statistics plug-in [47]. Direct and indirect effects between the dependent and independent variables as well as the mediator were tested with regression analyses (adjusted for significant abovementioned covariates), after which 95% CIs were calculated for the total indirect effects using bootstrapping (5,000 samples) as an unbiased means of testing whether the mediation model was valid. The presence of a mediation effect signifies that instead of having a direct causal effect between the independent variable (VO_2_ max) and dependent variable (IQ), the mediator (modular network topology) plays an important role in the association between these two variables.

## Results

### Subject characteristics

At this time-point in the AGHLS study, 344 healthy subjects participated. Our strict inspection of artifacts in the MEG recordings caused exclusion of 79 subjects. Fourteen subjects were excluded after examination of their intelligence scores, because they performed well below average (<75). Of the remaining 251 subjects, VO_2_max measurements were performed in 219 subjects, in whom all subsequent analyses were performed (see table 1 for subject characteristics). Subjects were on average 42 years old (range 41–44). With respect to IQ, men and women did not differ (t(217) = 1.005, p = 0.316). Men did have higher VO_2_ max (t(217) = 14.006, p<0.001) and greater head surface in cm^2^ (t(217) = 8.979, p<0.001). To ascertain that network topology results were not confounded by head surface size, this variable was used as a covariate in all analyses. Four MEG sensors were malfunctioning at the time of data collection, and these were excluded from further analysis in all subjects.

**Table 1:** Subject characteristics.

	Total group (N = 219)	Men (N = 106)	Women (N = 113)
Mean age in years (SD)	42 (0.7)	42 (0.7)	42 (0.7)
IQ score (SD)	108 (13)	109 (13)	108 (13)
Head surface in cm2 (SD)	231 (19)**	242 (17)	222 (15)
VO2max (SD)	46 (8.6)**	52 (7.0)	40 (5.5)

Note ** = p<.01, significant gender difference.

The last VO_2_ max measurement took place six years before MEG recording, when subjects were approximately 36 years old. In order to investigate whether this gap could induce large changes in physical fitness, we examined data from previous measurements in the AGAHLS cohort. These measurements were performed at 13, 14, 15, 16, 21, 27, 29 and 32 years of age in subgroups of the total cohort (with group sizes varying between 70 and 227 subjects). When looking at the consistency of VO_2_ max over these time points, there is strong consistency within subjects over time (see supplementary figure 1), with an average correlation coefficient R = 0.773 from one time point to the next. When comparing the first adult measurement at 21 years old and the measurement used in the remainder of this study at 36 years old (94 subjects overlapping), the correlation coefficient is 0.791. Furthermore, subjects who experienced major health burdens possibly influencing their lifestyle were excluded, which also ensures the stability and consistency of the VO_2_ max measurements up to MEG and IQ measurements six years later.

### Physical fitness, intelligence and brain modularity

The previously reported association between physical fitness and intelligence was confirmed: VO_2_ max was a significant predictor of intelligence in a linear regression model (B = 0.322, 95% CI [0.049 0.594], p = 0.021). We then set out to investigate our hypothesis concerning the association between physical fitness and brain network topology. Lower modularity in the upper alpha and beta bands was related to higher VO_2_ max (upper alpha band B = −1.81, 95% CI [−3.31 −0.315], p = 0.018; beta band B = −1.167, 95% CI [−1.753 −5.81], p = 0.017), adjusted for sex, head surface, and relative power per frequency band (see table 2 for results of all frequency bands).

**Table 2:** Associations between band-specific modularity and VO_2_ max.

	B	95% CI (B)	p-value
Delta band modularity	0.435	[-.080 0.167]	0.487
Theta band modularity	−0.390	[−1.64 0.086]	0.540
Loweralphamodularity	0.509	[−0.121 2.23]	0.561
Upperalphamodularity	−1.81	[−3.31 −3.15]	0.018*
Betamodularity	−1.17	[−1.75 −5.81]	0.017*
Gamma modularity	−11.1	[−80.9 58.6]	0.754

Note. *  =  p<0.05. Sex, relative power in each frequency band, and skull size were entered as covariates in each regression.

In order to confirm that these associations were indeed due to network topology instead of global connectivity levels, we performed an ANOVA with VO_2_ max as dependent variable and both modularity and average connectivity in the upper alpha and beta bands as independent variables. While the upper alpha and beta connectivity indices did not yield significant results (p = 0.529 and p = 0.869, resp.), modularity indices were significantly related to VO_2_ max (p = 0.016 and p = 0.012, resp.). The number of modules in the upper alpha and beta bands was not associated with VO_2_ max, indicating that it was not the number of modules that mattered, but the connectivity patterns within and between those modules.

### Modular connectivity

We then investigated the associations of between and within module connectivity with VO_2_ max in these two frequency bands. Results show that in the beta band, higher VO_2_max was related to increased between-module connectivity (B = 0.674, 95% CI [0.101 1.246], p = 0.021), indicating indeed that physical fitness is important for better intermodular integration. Moreover, an ANOVA with both between-module and average connectivity as covariates shows significant results for between-module connectivity only (p = 0.039 and p = 0.919, resp.), further underlining the added value of modularity-based connectivity over regular connectivity alone.

**Figure 2:**
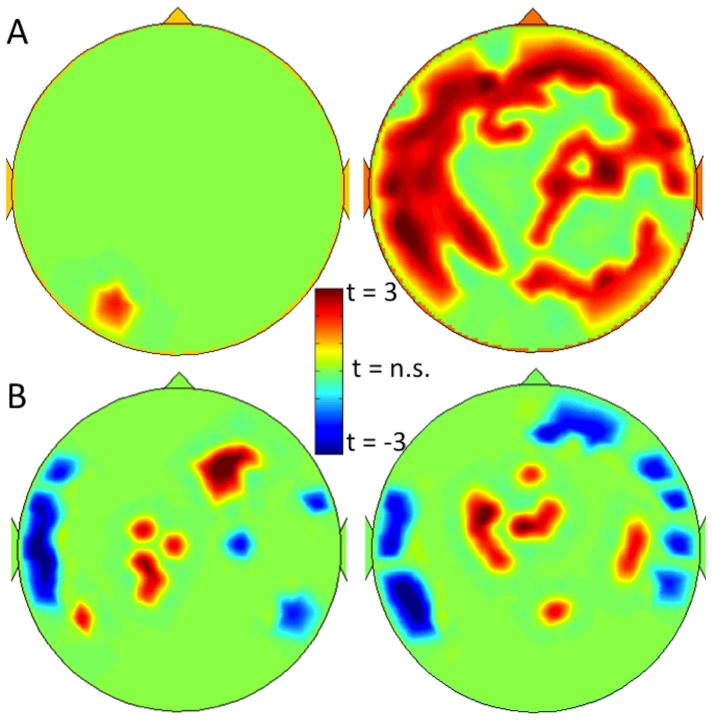
Significant sensor-specific associations between modular connectivity and VO_2_ max. Note. (a) shows an FDR-corrected t-map of significant associations between alpha band and beta band (left and right panel, resp.) between-module connectivity and VO_2_ max, while (b) shows the same for within-module connectivity. Warm colors indicate positive associations, cool colors refer to negative associations.

Figure 2a shows significant associations between VO_2_ max and between-module connectivity per channel for both upper alpha and beta bands, after correcting for the number of tests performed with the false discovery rate (FDR, q<0.05 [48]). These maps confirm the analysis of averaged between-module connectivity, and show positive correlations between VO_2_ max and between-module connectivity throughout the brain in the beta band, but not the upper alpha band.

Due to the nature of the within-module calculation (i.e. within-subject z-score is computed), no global average can be computed for this measure. However, figure 2b displays significant within-module connectivity associations with VO_2_ max in the upper alpha and beta bands, indicating that higher within-module connectivity in the central areas is positively associated with VO_2_ max, while the within-module connectivity within lateral temporal areas is negatively associated with physical fitness.

### Modularity and between-module connectivity as VO_2_ max – IQ mediators

**Figure 3:**
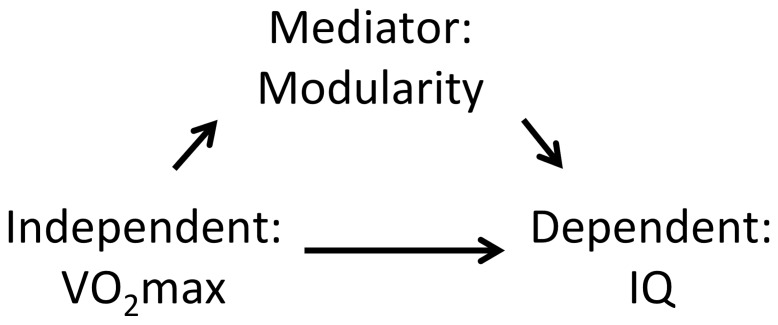
Graphical representation of hypothesized mediation effect. Note. A mediating effect of brain network topology on the association between VO_2_ max (maximum oxygen uptake during an effort test) and intelligence quotient (IQ) was hypothesized.

Finally, the associations between intelligence, VO_2_ max, and brain modularity were analyzed using mediation analyses. Our hypothesis was that better physical fitness leads to better cognitive performance and thus higher IQ later, through the mediating effect of brain network modularity (see figure 3). This hypothesis was not confirmed. Although separate regressions of the associations between both VO_2_ max and network characteristics and intelligence were significant, the mediation effects, as evidenced by significance levels and 95% confidence intervals through 5,000 bootstrapping samples, were not (see table 3). This indicates that although modularity, VO_2_ max and IQ are interrelated, the association between VO_2_max and intelligence is not statistically explained by modularity. Exploratory mediation analyses using different dependent, independent, and mediating variables also did not yield significant results.

**Table 3:** Mediation analyses of network topology on the association between physical fitness and intelligence.

Upper alpha band modularity (total 95% CI [−0.042 0.055])	Beta	p
VO2max - upper alpha modularity	−0.216	0.018*
VO2max - IQ total	0.187	0.046*
VO2max - IQ direct	0.185	0.052
Upper alpha band modularity mediation	−0.010	0.885
		
Beta band modularity (total 95% CI [−0.057 0.058])	Beta	p
VO2max - beta modularity	−0.197	0.018*
VO2max - IQ total	0.187	0.046*
VO2max - IQ direct	0.188	0.049*
Beta band modularity mediation	0.004	0.959
		
Beta band PC (total 95% CI [−0.032 0.074])	Beta	p
VO2max - beta PC	0.206	0.021*
VO2max - IQ total	0.187	0.046*
VO2max - IQ direct	0.184	0.053
Beta band PC mediation	0.013	0.854

Note. * p<0.05, CI  =  total confidence interval of indirect effects, based on 5,000 bootstrap samples.

Adjusted for sex, head surface, and relative band power. PC  =  participation coefficient.

## Discussion

Physical fitness and cognitive functioning are related. We show that this relation is also associated with topology of the functional brain network during the resting-state. Decreased upper alpha and beta band modularity were related to higher VO_2_ max, with higher beta between-module connectivity being associated with better physical fitness. Average functional connectivity did not show this association with VO_2_ max. The association between cardiorespiratory fitness and intelligence was however not statistically mediated by network characteristics.

Modularity refers to the extent to which the brain can be subdivided into coherent subsystems. Although such a modular organization is generally beneficial for brain functioning [35], having consistently tight connectivity within modules may be detrimental. Our results show negative correlations between modularity and both mental and physical functioning, indicating that higher levels of within-module connectivity versus between-module connectivity may be related to decreased functioning. An MEG study comparing modularity during several conditions of a working memory task reports decreasing modularity, i.e. increasing intermodular communication, as effort increases [49]. A study compiling a large number of task-related fMRI and PET studies also shows the importance of the modular organization of the brain for cognitive functioning [38]. However, how modularity relates to healthy functioning during the resting-state has not been reported.

Another resting-state study using modularity reports increased delta and theta band modularity in Alzheimer's patients when compared to healthy controls, which was related to poorer performance on a fluency task [37]. Furthermore, an fMRI study during task performance did not find changes in overall modularity over consecutive learning sessions, but does report that the flexibility of particular nodes, i.e. the number of times that each node in the network changes its belonging to specific modules, was related to better performance [50]. That is, having a highly dynamic modular structure, as opposed to a fixed modular division, was related to better functioning. These task-based findings concerning network flexibility have recently been replicated, localizing these multi tasking nodes mainly in the fronto-parietal network [51]. Our findings indicate that the resting-state is characterized by lower modularity and increased between-module, possibly long-range connections in brighter and fitter individuals. It would be interesting to investigate the transition from resting-state to any task, which may indicate that the resting-state modular flexibility of the brain network is similar to task-based dynamics.

The effects of modularity and between-module connectivity were present in the upper alpha and beta bands. These frequency bands havebeen studied extensively with respect to cognitive tasks, albeit mostly with respect to power and not connectivity or network properties. The (upper) alpha band has been related to attention and working memory [52]–[55], while the beta band has been implicated in learning, novelty detection, and reward evaluation, indicating that this oscillation might be an important mechanism for directing attention towards a novel stimulus [56]–[59]. A previous study used electroencephalography (EEG) to investigate connectivity and network efficiency during a task in active versus sedentary subjects [60]. Results show that in the beta band, active subjects show greater connectivity and network efficiency than sedentary subjects. Similar results were obtained when using coherence as a measure of connectivity, also in the alpha and beta band [61]. None of these studies investigated resting-state network topology.

With respect to the previously described study investigating modularity during increasingly difficult cognitive conditions [49], most effects of neural reconfiguration were found in the beta band, which the authors ascribe to the need for higher long-range synchronization, increased intermodular connectivity, and thus loss of modularity in this frequency band during tasks. This hypothesis, as well as our results, are corroborated by computational and animal work, showing that beta oscillations provide excellent support for long-distance synchronization [59], [62]. The beta band may speculatively be at the heart of communication between hub areas in the brain, which regulate higher-order functioning of the brain network and therefore relate to intelligence and cardio respiratory fitness, although more studies are needed to confirm this hypothesis.

Previous studies have only reported associations between resting-state functional connectivity, cognitive functioning, and physical fitness. Particularly higher connectivity within the default mode network (DMN) has been related to increased cardiorespiratory fitness, while DMN connectivity also mediates the association between VO_2_ max and cognitive functioning [17]. After a 1-year exercise intervention in older adults, both the DMN and the fronto-parietal network show higher connectivity than a control group [18], further building on the causal relationships that might exist between physical fitness and functional connectivity. Our results partly corroborate these findings, and indicate that there might be differential associations with particular types of connectivity: in our investigation of a very large cohort of healthy subjects with a direct measurement of neural activity, particularly increased between-module connectivity was related to superior cardiorespiratory fitness and intelligence. Also, our lack of findings with respect to average functional connectivity indicate that network analysis contributes valuable information to the association between fitness and intelligence, and advocates for investigation of the brain network as a whole instead of only focusing on connectivity between particular spatially determined areas.

Although circumstantial evidence is available, the definite direction of the association between increased cardio respiratory fitness, functional brain network organization, and cognition is still uncertain, and our results in a large sample do not support the hypothesis that better physical condition leads to better intelligence through brain network topology. Several aerobic intervention studies, which usually randomize between an exercise program and a control intervention of for instance light stretching, have reported increased cognitive functioning afterwards, but a number of studies failed to find a cognitive effect of increased cardio respiratory fitness [7], [63]. Our study was not aimed at addressing this issue, and mediation analyses were not significant. Additionally, measurement of VO_2_ max took place approximately six years prior to intelligence testing and MEG recording. Our analysis of VO_2_ max at previous time points suggests that this measurement is a relatively stable measure of physical fitness, and all subjects with disease burden influencing their lifestyle were excluded. Finally, the presence of associations between VO_2_ max and intelligence six years later suggest that we are indeed looking at a robust indication of physical fitness. However, we cannot ascertain that this interval between measurements did not influence our results. Future longitudinal studies are needed to shed light on the causal relations between cardiorespiratory fitness, intelligence, and network topology, while investigation of anatomical brain connections may also yield further insights into this issue.

Increased physical fitness is associated with better functional brain network topology. The step from exercise to functional brain network may be difficult to understand. On a cellular level, better physical fitness has often been associated with increases in BDNF [64], [65], and possibly with IGF-1 and VEGF [66]. A recent study suggests that these exercise-induced cellular changes are indeed related to functional connectivity, by comparing BDNF, IGF-1 and VEGF levels in two groups of participants undergoing either an aerobic or non-aerobic intervention [20]. The link between cellular biology and network functioning as measured with MEG has recently also been addressed in a study of protein expression and epilepsy in brain tumor patients [67]. We were able to show a direct association between epilepsy-related protein expression and between-module connectivity of the tumor area, further indicating that these network patterns may be the intermediate between molecules and behavior. Future studies are needed to further explore how cellular changes as a consequence of exercise lead to changes in functional connectivity.

Several limitations of the current study should be recognized. First of all, as previously mentioned, measurement of cardiorespiratory fitness was performed several years before MEG recording and intelligence testing took place. The influence of the lag between measurements in the current study design on the reported results is unknown. Secondly, this study was performed on the sensor-level, since no anatomical MRI scans (which are necessary to perform accurate source reconstruction in MEG data) were available. This limits the spatial specificity of our results, and prohibits further investigation of specific spatial network properties. Third, the spatial resolution of MEG is limited. Although MEG is much less sensitive to volume conduction and disturbing effects of the skull and scalp than EEG, common sources still pose a serious problem for coupling analysis. However, the phase lag index is a particularly strict measure of functioning connectivity, because it excludes all non-zero lagged correlations [44], [68].

In conclusion, we show that functional brain network organization may mediate the association between cardiorespiratory fitness and intelligence. Less tightly connected, more interconnected functional modular topology in the upper alpha and particularly beta band may promote long-range connectivity in the resting-state, which relates to both increased physical and mental fitness.

## Supporting Information

Figure S1 **Temporal consistency of VO_2_ max measurements in the AGAHLS cohort.** Note. (a) depicts the correlations between VO_2_ max measurements at each neighboring time point in the AGAHLS study, the first two measurements being performed at 13 and 14 years old (YO). The number of overlapping subjects between time points is indicated in parentheses. In (b), the first adult VO_2_ max measurement at 21 years old is correlated to the last measurement at 36 years old, which we used in this study (correlation coefficient of 0.791, p<0.001).
(TIFF)
