# Supplementary figures and images for: A Healthy Brain in a Healthy Body: Brain Network Correlates of Physical and Mental Fitness
Source: PLoS One. 2014 Feb 3;9(2):e88202. doi: 10.1371/journal.pone.0088202 (PMC3912221; doi:10.1371/journal.pone.0088202)

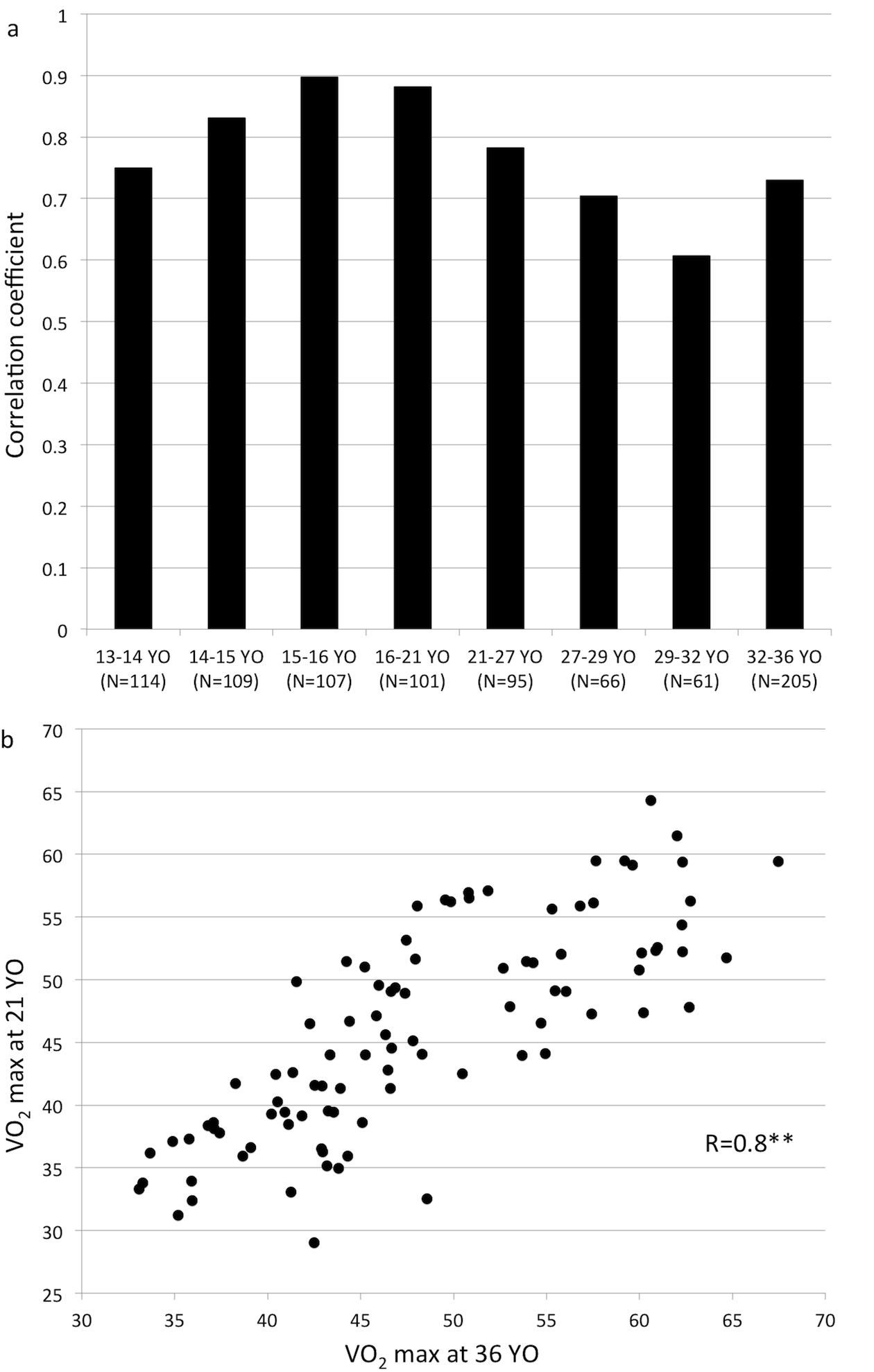

Supplement: Figure S1 — Temporal consistency of VO2 max measurements in the AGAHLS cohort. Note. (a) depicts the correlations between VO2 max measurements at each neighboring time point in the AGAHLS study, the first two measurements being performed at 13 and 14 years old (YO). The number of overlapping subjects between time points is indicated in parentheses. In (b), the first adult VO2 max measurement at 21 years old is correlated to the last measurement at 36 years old, which we used in this study (correlation coefficient of 0.791, p<0.001). (TIFF) [file pone.0088202.s001.tif]
